# Changes in serum levels of apelin and nitric oxide in hospitalized patients with COVID-19: association with hypertension, diabetes, obesity, and severity of disease

**DOI:** 10.1186/s40001-022-00852-3

**Published:** 2022-11-09

**Authors:** Farzaneh Rostamzadeh, Hamid Najafipour, Rostam Yazdani, Samira Nakhaei, Ahmad Alinaghi Langari

**Affiliations:** 1 Physiology Research Center, Institute of Neuropharmacology, Kerman University of Medical Sciences, Kerman, Iran; 2 Cardiovascular Research Center, Institute of Basic and Clinical Physiology Sciences, Kerman University of Medical Sciences, Kerman, Iran; 3 Endocrinology and Metabolism Research Center, Institute of Basic and Clinical Physiology Sciences, Kerman University of Medical Sciences, Kerman, Iran; 4 Gastroenterology and Hepatology Research Center, Kerman University of Medical Sciences, Kerman, Iran

**Keywords:** Apelin, Nitric oxide, COVID-19, Hypertension, Diabetes mellitus, Obesity

## Abstract

**Background:**

COVID-19 is an infectious disease currently spreading worldwide. The COVID-19 virus requires angiotensin-converting enzyme 2, an enzyme that plays a vital role in regulating the apelinergic system for entry into target cells. The underlying diseases of hypertension, diabetes mellitus, and obesity are risk factors for the severity of COVID-19 infection. This study aimed to compare the serum levels of apelin and nitric oxide in hospitalized COVID-19 patients and non-COVID-19 subjects with and without the mentioned risk factors.

**Methods:**

Serum samples were taken from 69 COVID-19 patients and 71-matched non-COVID-19 participants enrolled in the Kerman coronary artery disease risk factors cohort study. Study participants were divided into eight groups of control (healthy), hypertension, diabetes mellitus, obesity, COVID-19, COVID-19 + hypertension, COVID-19 + diabetes mellitus, and COVID-19 + obesity (*n* = 15–20 in each group). Serum apelin and nitrite were measured by the enzyme-linked immunosorbent assay and colorimetric methods, respectively.

**Results:**

Hypertensive and obese patients had lower serum apelin compared to the control group. In addition, apelin content was lower in the COVID-19 and COVID-19 + diabetes mellitus groups compared to the non-COVID-19 counterpart groups. Serum apelin levels were positively associated with arterial O_2_sat. and negatively with the severity of lung involvement. Nitric oxide metabolites were significantly lower in the COVID-19, COVID-19 + diabetes mellitus, and COVID-19 + obesity groups.

**Conclusions:**

The lower apelin and nitric oxide levels in patients with hypertension and obesity or their reduction due to infection with COVID-19 or concomitant COVID-19 + diabetes mellitus may make them vulnerable to experiencing severe diseases.

## Introduction

The COVID-19 infection caused by the severe acute respiratory syndrome coronavirus 2 (SARS-CoV-2) was originally reported as an epidemic in Wuhan, China, in December 2019 [1]. This infection has spread worldwide and, according to the World Health Organization (WHO), is a health challenge sparking international alarm [2]. People with risk factors such as hypertension, diabetes mellitus, obesity, and other cardiovascular diseases (CVDs) risk factors, and those older than 55 years are at greater risk [3, 4]. As a result, taking care of the elderly and those with underlying CVDs is a higher priority, as more severely ill patients will impose higher expenses on the country’s healthcare system [5].

For entry into its target cells, the SARS-CoV-2 binds to the angiotensin-converting enzyme type 2 (ACE2) through glycoprotein spikes. ACE2, a member of the renin–angiotensin–aldosterone system (RAAS), is responsible for inactivating angiotensin I and II and producing angiotensin 1–7 [6].

Apelin originated from a 77-amino acid pre-propeptide with several functional isoforms, including apelin-36, apelin-17, apelin-13, and (Pyr1) apelin-13, which is a post-translationally modified isoform in the circulation [7]. Apelin and ACE2 have a bilateral relationship. ACE2 degrades the apelin peptides to inactive forms, while apelin increases ACE2 expression [8]. Apelin and its receptor (APLNR, also known as APJ) are expressed in many tissues, such as the heart, lung, and blood vessels, regulating the cardiovascular and respiratory systems [9–11]. Apelin reduces arterial blood pressure by increasing nitric oxide (NO) generation and enhancing heart contractility. This peptide also protects the heart against ischemic–reperfusion injuries [11] and has antithrombotic properties [9]. Apelin also improves pulmonary hypertension and is a protective factor against acute respiratory distress syndrome (ARDS) [10]. Its circulatory concentration changes in diabetes mellitus, hypertension, metabolic syndrome, and obesity [12–16], the diseases that aggravate the severity of COVID-19 [3, 4, 17].

Given the regulatory roles of apelin/APJ in the cardiovascular system and its relation with ACE2 and COVID-19 patients’ symptoms, which are characteristic of dysregulation of apelin/APJ signaling pathways, such as ARDS, vascular dysfunction, thrombosis, and inflammation [11, 18, 19], this study aimed to evaluate the serum levels of apelin in patients with COVID-19 with and without the underlying diseases of hypertension (HTN), diabetes mellitus (DM), and obesity (OB). Considering that some effects of apelin, such as the antithrombotic and antihypertensive effects, are exerted through the NO signaling pathway, the amount of NO metabolites in the serum of these patients was also evaluated.

## Materials and methods

In this case–control study, 69 COVID-19 subjects were selected from patients who were referred to and hospitalized at Afzalipour Hospital in Kerman, Iran, the main referral center in the city, from February to November 2020. Seventy-one non-COV matched subjects were selected from individuals who were ordinary city residents who participated simultaneously in the Kerman coronary artery disease risk factors study (KERCADRS) phase III [20].

COVID-19 infection was confirmed by positive reverse transcription–polymerase chain reaction (RT–PCR). The clinical and lung computed tomographic scan (CT-scan) findings were recorded after admission and interpreted by the physicians. The current smoking history and opium use were recorded by interview. The severity of the COVID-19 disease was almost similar on the first day of admission in all patients when the blood samples were taken. The blood was centrifuged at 4000 g, and serum was stored at − 80 °C.

The hypertensive subjects were those with systolic blood pressures equal to or above 140 mm Hg and/or diastolic blood pressure equal to or above 90 mm Hg or those taking antihypertensive drugs [21]. Those with diabetes mellitus were selected from subjects whose fasting blood sugar was higher than 126 mg/dL or who were taking anti-diabetic drugs [22]. People with a BMI equal to or above 30 kg/m^2^ were considered obese [23]. The hypertensive and diabetic patients with COVID-19 were chosen based on their history of having these underlying diseases as COVID-19 increases the possibility of hyperglycemia and hypertension after infection [24].

The participants were divided into eight groups: 1—Control (CTL, *n* = 20), healthy individuals who did not have COVID-19 or any underlying disease, 2—HTN (*n* = 20), 3—DM (*n* = 17); 4—OB (*n* = 15), 5—COVID-19 (COV) (*n* = 20), patients with COVID-19 who did not have any underlying disease, 6—COV + HTN (*n* = 17), 7—COV + DM (*n* = 15), and 8—COV + OB (*n* = 17). The number of subjects in the groups was chosen according to similar previous studies [25]. The participants’ demographic and initial clinical characteristics are presented in Table 1. We did our best for the corresponding case and control groups to be matched based on sex, age, body mass index (BMI), and history of diabetes, hypertension, and hyperlipidemia.

**Table 1:** Clinical and demographic findings of the studied groups

	CTL	HTN	DM	OB	COV	COV + HTN	COV + DM	COV + OB
Number	20	20	17	15	20	17	15	17
Male (%)	10 (50)	8 (40)	9 (52.9)	9 (60)	10 (50)	9 (52.9)	6 (40)	11 (64.7)
Age (years)	54 (20)	61 (18)^a^	50 (26.7)	43 (25)	63 (17.2)	67 (23)^a^	59 (14)	43 (14.7)
BMI (Kg/m^2^)	21.6 (2.7)	22.4 (2.43)	22 (4.2)	30.5 (2.1)^c^	22.4 (3.8)	24.3 (4.3)b	26 (4.4)^b^	31.15 (4.1)^c^
SBP (mmHg)	120 (20)	150 (23)c	114 (10)	110 (10)	130 (28)^a^	147 (35)^c^	130 (42)^bd^	130 (21)^a^^e^
DBP (mmHg)	70 (20)	90 (23)^c^	70 (10)	75 (10)	77 (18)	82 (22)^b^	79 (13.7)^b^	80 (16.5)^b^
DM history	No	No	Yes	No	No	No	Yes	No
HL history	No	No	No	No	No	No	2 (13.3℅)	No
HTN history	No	Yes	No	No	No	Yes	No	No

The data are in *n* (%) or as median (IQR) the categorical variables were analyzed using the chi-square test. The continuous data were analyzed by Kruskal–Wallis test followed by Mann–Whitney *U* test

*CTL* control (healthy people), *COV* COVID-19, *HTN*: hypertension, *HL* hyperlipidemia *DM* diabetes mellitus, *OB*: obesity, *SBP*: systolic blood pressure, *DBP*: diastolic blood pressure

^a^*P* < 0.05

^b^*P* < 0.01

^c^*P* < 0.001 vs. CTL group

^d^*P* < 0.05 vs. DM group

^e^*P* < 0.05 vs. OB group

## Measurement of biochemical factors

The level of serum apelin-13 was determined by the enzyme-linked immunosorbent assay (ELISA) method according to the instructions of the kit (Bioassay Technology Laboratory, China). Serum NO content was evaluated by measuring its metabolite nitrate using the Rice calorimetric method. In this method, nitrate is converted to nitrite by vanadium (III) chloride (VaCl3), and the optical density (OD) is measured at 540 nm [26].

## Statistical analysis

Data in the tables and figures are presented as median with interquartile range (IQR) for continuous and *n* (%) for categorical variables. The continuous variables were analyzed using the Mann–Whitney *U* test for two independent groups (e.g., for the COV with non-COV groups) and the Kruskal–Wallis test for comparison among different study groups (CTL, HTN, DM, OB, COV, COV + HTN, COV + DM, and COV + OB), accompanied by the Mann–Whitney *U* test. The categorical variables were analyzed using the chi-square test. Multivariable linear regression was used to assess the correlation between duration of hospitalization and arterial O_2_sat. with apelin levels. The models were adjusted for sex, age, BMI, hypertension, diabetes status, and other covariates. Pearson correlation test was used to assess the association between apelin levels and other variables, such as age, arterial O_2_ saturation, and NO levels. *P* values < 0.05 were considered significant.

## Results

The demographic data of the studied groups are summarized in Table 1. The sex, age, and hyperlipidemia status of the subjects are matched among the groups. Only HTN groups have significantly higher ages than the CTL group (*P* < 0.05). The arterial systolic and diastolic blood pressure of the COV, HTN, COV + HTN, COV + DM, and COV + OB groups were higher than the CTL group (*P* < 0.05 to *P* < 0.001). However, in the non-HTN groups blood pressure was below the threshold level for hypertension. As expected, BMI in the OB and COV + OB groups was also higher than in the CTL group (*P* < 0.001). BMI was also higher in the COV + DM and COV + HTN groups than the CTL group (*P* < 0.01), although it was below the defined level for obesity.

**Fig. 1:**
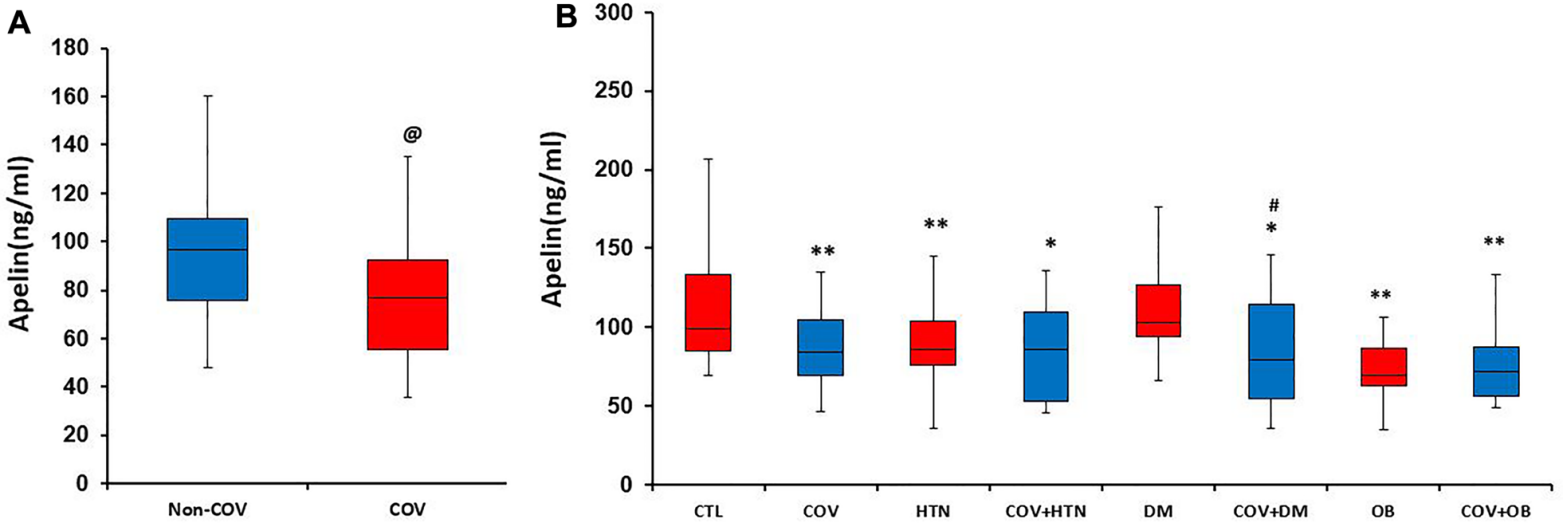
Apelin level in studied groups. Panel **A** indicates overall apelin level in COV patients compared to non-COV individuals. Analyzed by Mann–Whitney *U* test. Panel **B** indicates apelin level based on underlying diseases subgroups, analyzed by Kruskal–Wallis test followed by Mann–Whitney *U* test. @ *P* < 0.05 *vs*. non-COV, *P < 0.05, **P < 0.01, *** *P* < 0.001 vs. CTL and, #*P* < 0.05 vs. DM group. *CTL* control (healthy subjects), *HTN* hypertension, *DM* diabetes mellitus, *OB* obese. *n* = 15–20 in each group

Overall, the apelin levels were significantly lower in COV patients compared to non-COV participants (*P* < 0.05) (Fig. 1A). They were also lower in patients with COVID-19 without any underlying disease than healthy individuals (*P* < 0.01). Apelin levels in patients with COV + DM were lower than in the CTL and DM groups (*P* < 0.05). As apelin levels were already low in the HTN and OB groups, infection with SARS-CoV-2 did not reduce them further (Fig. 1B).

**Fig. 2:**
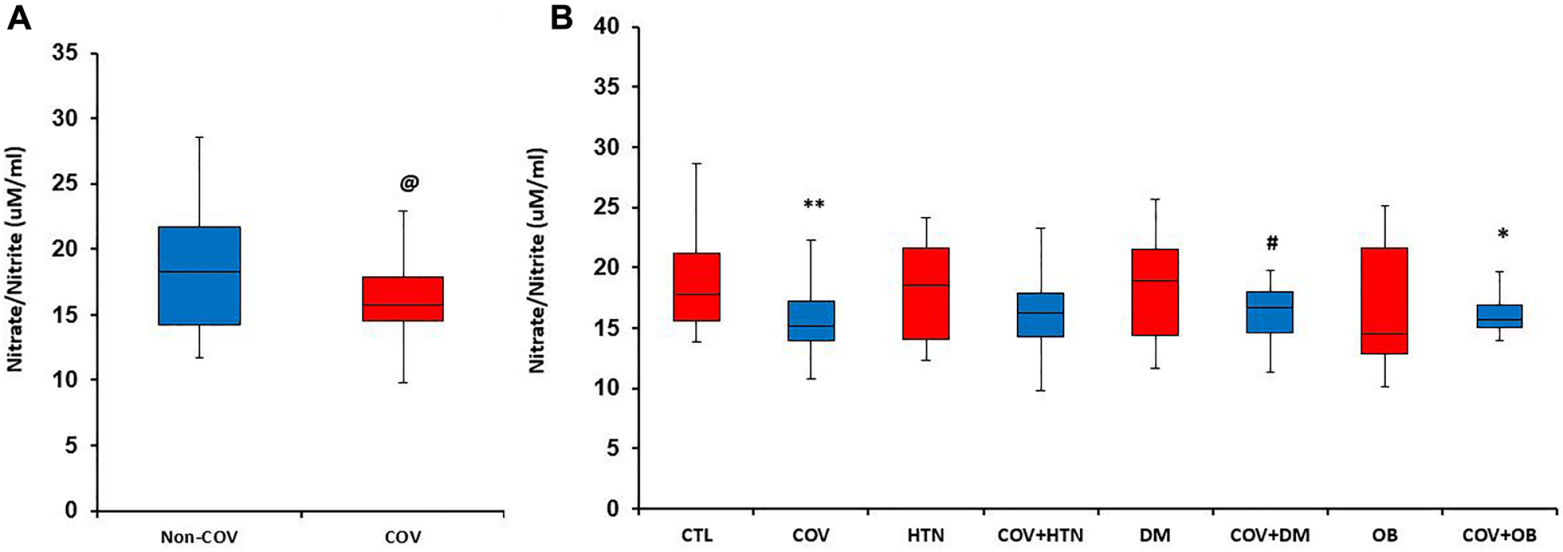
Nitrite level in the studied groups. Panel **A** indicates overall nitrite level in COV patients compared to non-COV individuals analyzed by Mann–Whitney *U* test. Panel **B** indicates nitrite level based on underlying diseases subgroups analyzed by Kruskal–Wallis test followed by Mann–Whitney *U* test. @ *P* < 0.05 vs. non-COV, *P < 0.05, **P < 0.01, vs. CTL. #*P* < 0.05 vs. DM group, *CTL* control (healthy subjects), *HTN* hypertension, *DM* diabetes mellitus, OB obese. *n* = 15–20 in each group

Overall, nitrite levels (NO metabolite) were significantly lower in COV compared to non-COV subjects (*P* < 0.05) (Fig. 2A). In addition, in people with non-underlying diseases they were significantly lower in the COV subgroups compared to the non-COV subgroups (*P* < 0.05). In the COV + OB group, nitrite levels were significantly lower compared to the CTL group (*P* < 0.05) (Fig. 2B). Furthermore, nitrite levels were lower in the COV + DM group than the DM group (*P* < 0.05).

Serum apelin levels in COVID-19 patients were not dependent on sex, smoking status, and opium use (Table 2). The findings also indicated that apelin levels were lower in patients who had more extensive lung involvement, according to their CT scans (*P* = 0.03) (Table 2).

**Table 2:** Association between apelin levels and sex, smoking, opium use and the severity of lung involvement in COVID-19 patients. The data are in Median (IQR)

	Apelin (ng/ml)	*P* value
Sex		
Male (54.5%)	81.1 (39.6)	
Female	84.4 (63)	0.3
Opium use		
Yes (24.8%)	61.6 (55.2)	
No	84 (49)	0.09
Smoking		
Yes (10.7%)	69.1 (50)	
No	82 (50)	0.2
Chest CT		
Negative (31.4%)	90.2 (59)	
With ground-glass change (31.4%)	80 (46)	0.05
With consolidations (37.1%)	79.4 (38)	0.01

The difference among groups was analyzed by Kruskal–Wallis and Mann–Whitney *U* tests

**Fig. 3:**
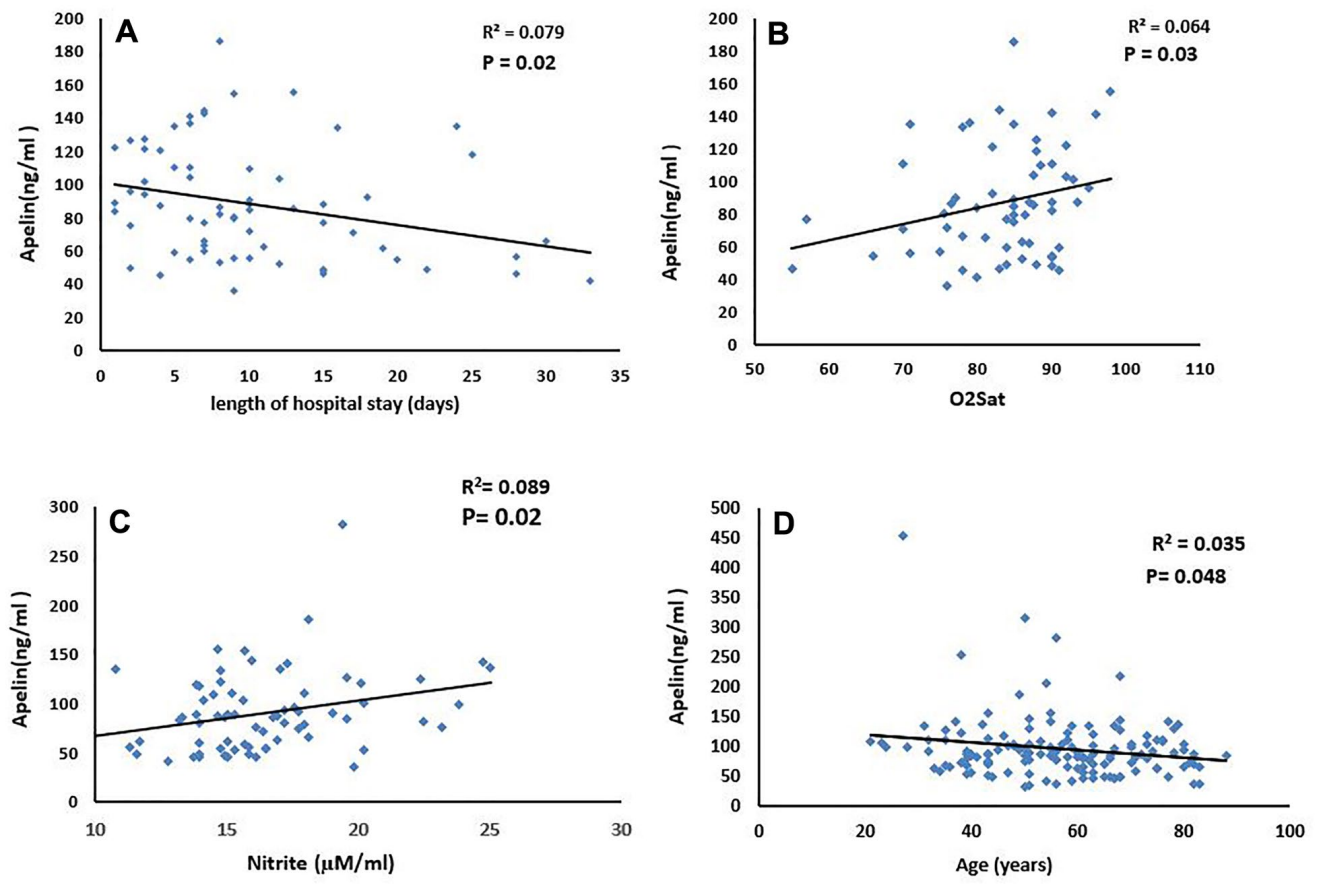
Pearson correlation test indicated correlations between serum apelin level with length of hospital stay **A** and arterial O_2_ saturation **B** in COV patients and the level of serum nitrite **C** and age **D** in studied groups. O_2_Sat: arterial O_2_ saturation

The association of apelin levels with disease severity according to the length of hospital stay and arterial O_2_ saturation was analyzed using multivariable linear regression. There was no significant relationship between duration of hospitalization and arterial O_2_ saturation with age, sex, BMI, DM, and HTN status (Table 3). However, there was a negative linear relationship between serum apelin levels and the length of hospital stay (*R*^2^ = 0.078 *P* = 0.02) (Fig. 3A and Table 3). There was a positive correlation between apelin levels and O_2_ saturation (*R*^*2*^ = 0.064 *P* = 0.03) (Fig. 3B and Table 3). The results also showed a positive linear association between serum apelin and nitrite levels in the studied subjects (*R*^*2*^ = 0.089 *P* = 0.02) (Fig. 3C). A negative association between age and apelin serum levels was also observed (*R*^2^ = 0.035 *P* = 0.048) (Fig. 3D).

**Table 3:** Multiple linear regression of length of hospital stay and arterial O_2_ saturation, as dependent variable, with age, BMI, DM, HTN, sex and serum apelin level in studied groups

Dependent Variable	Predictors	Beta	*P* value
Length of hospital stay	BMI	− 0.023	0.888
	Age	0.124	0.402
	HTN	0.094	0.493
	DM	− 0.177	0.189
	Sex	− 0.192	0.167
	Apelin level	− 0.284	0.040
Arterial O_2_ saturation	BMI	− 0.177	0.245
	Age	− 0.285	0.079
	HTN	0.056	0.697
	DM	− 0.067	0.633
	Sex	0.041	0.765
	Apelin level	0.413	0.004

## Discussion

The findings of this study showed that the apelin and nitric oxide content was lower than normal in patients with COVID-19 on admission. However, apelin levels were already decreased in patients with underlying risk factors, including hypertension and obesity, and remained low after infection with SARS-CoV-2. The reduction of serum apelin concentration associated with the reduction of O2 Sat. and the extent of lung involvement could partly predict the severity of diseases.

Apelin is a protective peptide in the cardiovascular and respiratory systems, and it exerts its beneficial effects through its receptor, APJ [10]. Apelin and its receptor are present in vascular endothelial cells, including lung blood vessels [10].

Apelin has also been identified as a substrate for ACE2. ACE2 degrades and inactivates some apelin isoforms in a process similar to its primary substrate, Ang-II [19]. Considering that SARS-CoV-2 reduces the amount and/or activity of ACE2 by binding to it, it is predictable that the level of apelin is reduced in COVID-19 patients. This study's findings showed that the serum apelin content was significantly lower in control and diabetic people with COVID-19, and the amount of its reduction levels were positively associated with the hospital duration. Therefore, it is suggested that lower apelin levels may worsen COVID-19 disease complications. Apelin can exert its effect directly by binding to APJ and acting on several signaling pathways in different tissues, including the respiratory and circulation system, and indirectly by enhancement of ACE2 expression [27, 28]. In experimental models of ARDS, apelin inhibits pro-inflammatory cytokine production, reduces inflammation, and improves oxygenation by activation of AKI–eNOS pathways [29]. The lower serum apelin levels found in COV patients in this study can be a result of its degradation by ACE2 as circulatory ACE2 has been shown to be increased in patients with COVID-19 [30]. In an animal model of ARDS, there was a significant decrease in the level of apelin in blood and lung tissue [29], and ACE2 and apelin expressions decreased in cardiomyocytes infected by SARS-CoV-2 [27].

The regulatory role of apelin in ACE2 gene expression has been proven [28]. Apelin enhances the transcription of the ACE2 gene in the rat heart [31] and in cardiomyocytes and adipose tissues of diabetic mice [28]. The enhancement of ACE2 expression could activate the signal pathways of ACE2/Angiotensin-II/Angiotensin-[1–7], which leads to the diminution of the detrimental effects of Ang-II. Therefore, the use of apelin or its analogs may be recommended for the treatment of COVID-19 patients. The concern is that apelin may increase the rate of SARS-CoV-2 entry into the cells by increasing the expression of ACE2 [8]. However, recent findings have shown that ACE inhibitors and the angiotensin type 1 receptor (AT1R) blockers do not increase the susceptibility to viral infection and the severity of COVID-19 [33]. Currently, these findings are contradictory, and this subject requires further investigation.

Our findings indicated that apelin levels are positively associated with O_2_ saturation. In contrast, tissue hypoxia and acute systemic hypoxia enhance apelin and APJ expression in the heart, kidney, and brain by inducing hypoxia-inducible factor-1 (HIF-1) and increasing circulating apelin levels in humans and animals [15, 34]. Treatment with apelin decreases the injuries induced by hypoxia in different organs. It seems that in normal conditions increase in apelin production is a compensatory mechanism to counter the harmful effects of hypoxia. However, in diseased conditions, such as COVID-19 infection, more ill people already have a lower level of apelin and lower O_2_sat.

The results of the present study also revealed that NO levels were lower in COVID-19 patients, as were apelin levels. Some of the effects of apelin, such as vasodilation and anticoagulant effects, are mediated by NO [11, 18]. Therefore, it was not unexpected that the concentration of NO decreased along with the reduction of apelin levels.

The finding that the amount of apelin decreases with increase in age may be consistent with the higher rate of complications and severity of COVID-19 in older patients (because of the reduction in protective effects of apelin). Apelin also has a role in regulating blood glucose by increasing insulin sensitivity [35]. Decreased apelin concentration in COVID-19 patients may be one of the reasons for the disturbances in blood glucose regulation in these patients, even in those who are without diabetes mellitus. The reduction of apelin in subjects with diabetes mellitus can worsen blood glucose regulation in these patients when they are infected with COVID-19. Moreover, our results indicated that in subjects with hypertension and obesity, apelin levels are also lower than normal. That is why obesity and hypertension are also among the comorbidities of COVID-19. These patients are also more likely to get COVID-19 and progress to ARDS when infected with the SARS-CoV-2 virus [3, 36].

We acknowledge that it would have been better if the sample size in each group had been larger. The small sample size was due to the limitations of finding subjects with only one of the underlying diseases while being matched for age, sex, and BMI to reduce the confounding factors that may affect serum apelin levels. In addition, we measured the levels of apelin and NO in subjects who were hospitalized. The levels of mentioned factors may vary in outpatient who had mild type of the disease.

## Conclusions

We found lower apelin and nitric oxide levels in patients with hypertension and obesity and in their COVID-19-infected counterparts. Since arterial O_2_ saturation, hospitalization period, and degree of lung involvement were closely associated with serum apelin levels, this factor may predict the severity of the disease, especially in those with underlying cardiovascular risk factors, such as diabetes mellitus, obesity, and hypertension.

## Data Availability

Data will be available on request

## Abbreviations

ACE2: Angiotensin-converting enzyme type
APLNR: Apelin receptor
ARDS: Acute respiratory distress syndrome
AT1R: Angiotensin type 1 receptor
BMI: Body mass index
CTL: Control
CT-scan: Computed tomographic scan
CVDs: Cardiovascular diseases
DM: Diabetes mellitus
ELISA: Enzyme-linked immunosorbent assay
HIF-1: Hypoxia-inducible factor-1
HTN: Hypertension
IQR: Interquartile range
NO: Nitric oxide
O_2_sat: Arterial O_2_ Saturation
OB: Obesity
RAAS: Renin–angiotensin–aldosterone system
RT–PCR: Reverse transcription–polymerase chain reaction
SARS-CoV-2: Severe acute respiratory syndrome coronavirus 2
WHO: World health organization
